# Individualizing Representational Similarity Analysis

**DOI:** 10.3389/fpsyt.2021.729457

**Published:** 2021-10-11

**Authors:** Seth M. Levine, Jens V. Schwarzbach

**Affiliations:** ^1^Institute of Cognitive and Clinical Neuroscience, Central Institute of Mental Health, Medical Faculty Mannheim, Heidelberg University, Mannheim, Germany; ^2^Department of Psychiatry and Psychotherapy, University of Regensburg, Regensburg, Germany

**Keywords:** fMRI, individual differences, multivariate pattern analysis, precision psychiatry, representational similarity analysis, task-based imaging

## Abstract

Representational similarity analysis (RSA) is a popular multivariate analysis technique in cognitive neuroscience that uses functional neuroimaging to investigate the informational content encoded in brain activity. As RSA is increasingly being used to investigate more clinically-geared questions, the focus of such translational studies turns toward the importance of individual differences and their optimization within the experimental design. In this perspective, we focus on two design aspects: applying individual vs. averaged behavioral dissimilarity matrices to multiple participants' neuroimaging data and ensuring the congruency between tasks when measuring behavioral and neural representational spaces. Incorporating these methods permits the detection of individual differences in representational spaces and yields a better-defined transfer of information from representational spaces onto multivoxel patterns. Such design adaptations are prerequisites for optimal translation of RSA to the field of precision psychiatry.

## Introduction

Over the past decade, the multivariate method of Representational Similarity Analysis (RSA) (1) has become a popular means of investigating the informational content represented within patterns of activity in the brain. This technique allows researchers to test hypotheses regarding the relative high-dimensional structure of information, represented by multivariate activity patterns, in different regions of the brain. Since its development, RSA has been used with functional neuroimaging to explore topics such as object categorization (2), semantics (3), object recognition (4), affect (5), action observation (6), and fear-conditioning (7). Studies employing such multivariate analyses have provided the field with an improved understanding of how information is encoded in local activity patterns and how such high-dimensional information can be altered through processes such as attention (8) or aversive-learning (9–11). Recently, researchers have begun translating these methods to psychiatry in an attempt to explore differences in representational spaces between individuals with and without disorders (12–16).

While the discipline of cognitive neuroscience has tended to use functional magnetic resonance imaging (fMRI) to explore group-level effects in mapping functions to brain structures, there has been a recent push to investigate individual differences using fMRI (17–19) and to incorporate advanced analysis methods into precision psychiatry (20, 21). As such, individualizing neuroimaging analyses is a critical step for studying individual differences in human behavior, which can be facilitated by (a) preserving a given individual's behavioral data prior to carrying out RSA and (b) increasing the congruency between the conditions under which the behavioral and neuroimaging data are acquired. Below we provide a brief overview of RSA, describe these two strategies in further detail, and discuss aspects of the experimental design that researchers and clinicians can implement in (translational) work that focuses on individual differences.

## Representational Similarity Analysis: An Overview

Representational similarity analysis falls within the framework of multivariate pattern analysis (22–24), which investigates the activity from many distributed neurons as a function of mental representations. Rather than using the *average* activity of such neurons (i.e., the classic univariate approach) as the proxy for mental representations, multivariate analyses take advantage of the *pattern* of activity across voxels, thereby providing a more informative approach to exploring and making inferences about representational spaces [i.e., abstract spaces of mental representations within the cognitive architecture whose structures are defined by some feature(s)]. Extending this logic, RSA specifically operationalizes *dissimilarities* in representational spaces as the dissimilarities between evoked multi-voxel patterns and then compares this dissimilarity structure (i.e., the set of neural dissimilarities in question) to a behavioral or theoretical dissimilarity structure. As this approach depends on relative differences between voxels (rather than differences within voxels), RSA provides researchers with a richer means of uncovering higher-dimensional information within the structure of mental representations.

To carry out RSA with fMRI, researchers design a task-based study, in which stimuli are usually presented to participants in a trial-by-trial manner. During data analysis, one generally performs the classic mass-univariate analysis of fitting stimulus-specific models of the hemodynamic response to time course data evoked by those stimuli using the general linear model, and the resulting beta-weights (or t-scores) serve as the input for the multivariate analysis. One of the most common uses of RSA involves employing models that describe the relationship between stimuli, according to some organizing principle, and observing the extent to which a given model explains differences between the observed stimulus-evoked multivariate patterns in the brain.

**Figure 1:**
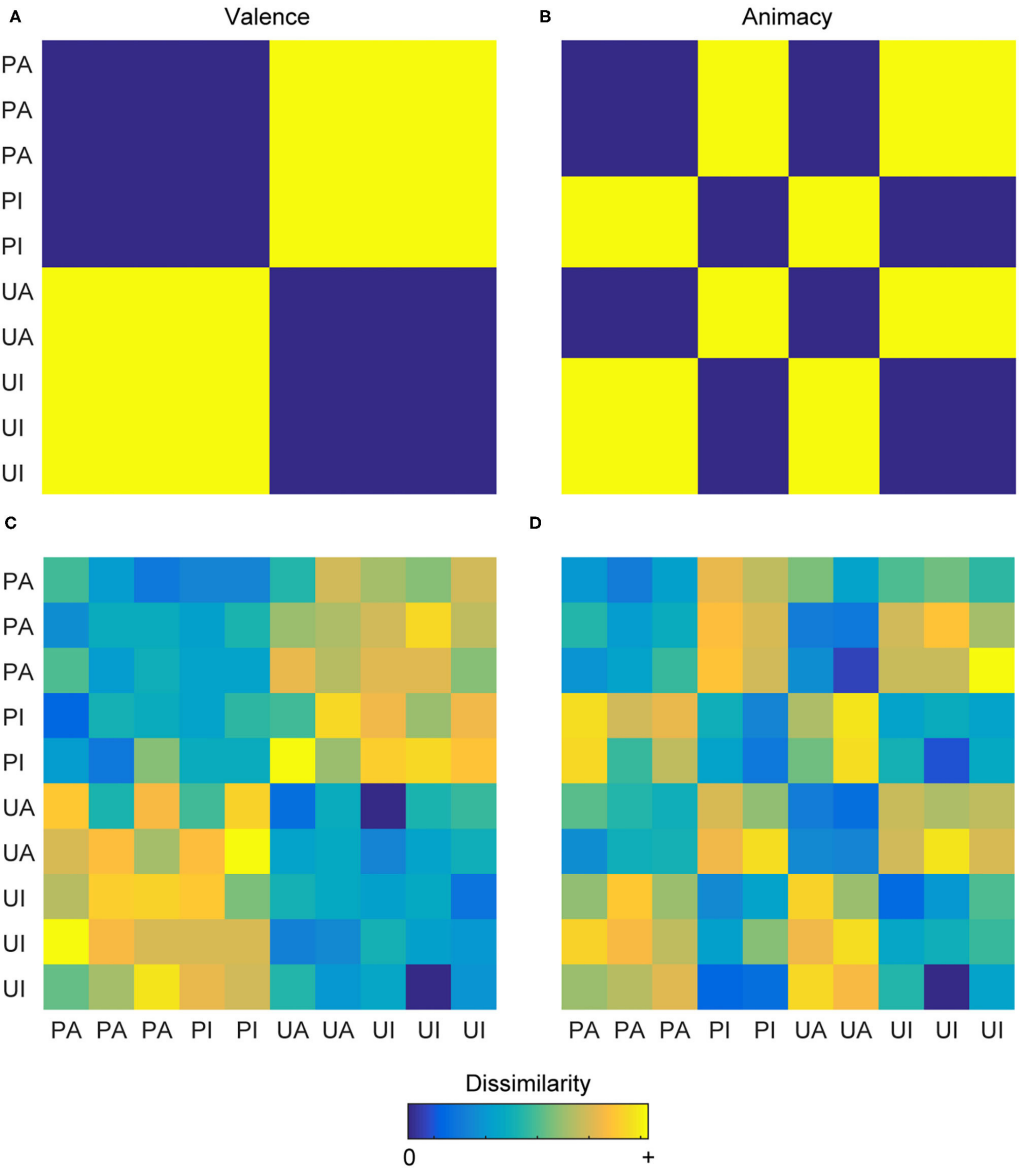
Representational dissimilarity matrices. **(A)** A theoretical RDM depicting the binary relationship between pairs of images classified as pleasant-animate (PA), pleasant-inanimate (PI), unpleasant-animate (UA), and unpleasant-inanimate (UI) according to their valence. **(B)** A theoretical RDM depicting the binary relationship between these same images according to their animacy. These two models are effectively uncorrelated (*r* = −0.08) and can be used to explore the hypotheses of whether a particular brain region encodes valence information or animacy information. Cool colors depict decreasing dissimilarity, and warm colors depict increasing dissimilarity. **(C,D)** The same conventions as the upper row but depicted here are two RDMs that might result from a participant having carried out the multi-arrangement method, yielding a similar information structure as the theoretical RDMs but additionally containing graded, individualized information.

For example, if presenting participants with 10 pictures (five pleasant pictures and five unpleasant pictures), a researcher could develop a model of emotional valence in which the five pleasant pictures are considered similar to each other, the five unpleasant pictures are considered similar to each other, and the pleasant and unpleasant pictures are considered dissimilar to one other. This hypothesis can be visualized as a representational dissimilarity matrix (RDM), which is the relative dissimilarity structure reflecting the relationship between all pairs of stimuli under investigation (Figure 1A). An alternate model using the same images could see the stimuli organized according to their animacy. If some of the pleasant and unpleasant pictures contain people in them, these stimuli could be considered similar, and perhaps the remaining stimuli contain only inanimate objects, which could also be considered similar. The animate and inanimate stimuli would then be considered dissimilar to one another (Figure 1B). Pitting these two model RDMs against each other allows a researcher to determine whether the activity patterns in some brain region are better explained by the “valence” model and whether the activity patterns in some other brain region are better explained by the “animacy” model. The pairwise relationships between stimuli at the neural level are generally operationalized by considering their evoked multivoxel patterns (e.g., within a local neighborhood, a given brain region, or a set of brain regions) and computing a distance measure, such as Euclidean or correlation distance (25), between them. It is this *neural* RDM that the model RDMs seek to explain. Comparing the explanatory power of each model (or a combination thereof) with that of the other models allows researchers to determine which of the tested models best explains the representational space underlying the activity patterns and, consequently, interpret information processing at some level of the cognitive architecture.

Recently, RDMs have been generated through behavioral experiments, for example, by combining the multi-arrangement method (26) with inverse multidimensional scaling (27), rather than directly hypothesized by the researcher. During the multi-arrangement method, participants drag-and-drop stimuli into a 2D arena on a computer screen, in which the distances between stimuli reflect the participants' dissimilarity judgments of the stimuli, according to some organizing principle. Inverse-multidimensional scaling then estimates the RDM from the 2D inter-stimulus distances (Figures 1C,D). For additional details, see (27). These methods provide the benefit of yielding high-dimensional representational spaces that contain nuanced, idiosyncratic, and continuous information and have been used to generate *behavioral* RDMs in combination with RSA to investigate domains such as similarity of body parts (28), emotions (29), tool-related features (30), action observation (31), and face perception (32). A variety of tutorials, reviews, and methodological guides on RSA have been compiled over the past years for researchers seeking more extensive explanations of these concepts and current practices (25, 33–38).

Among these various domains, one of the manners in which RSA has proven to be particularly powerful is that it can yield comparable RDMs across participants, even when the evoked patterns themselves are not comparable [i.e., a second-order isomorphism (38)], thereby revealing a similar underlying representational structure across different individuals. On the other hand, also the *differences* between individuals' RDMs may be indicative of meaningful individual differences when interpretable through an additional variable (e.g., personality traits). As such, similar brain regions among participants can be investigated in terms of the individual differences in their underlying representational spaces. In the next sections we expand further on these points with respect to methodological strategies.

## Averaging vs. Preserving Individual RDMs

One approach to RSA involves researchers averaging behavioral RDMs from many participants and then comparing this averaged behavioral RDM to distinct individuals' neural RDMs. This approach yields one behavioral RDM per condition for all participants in the study, which fits the central paradigm of general psychology to test for commonalities among samples: i.e., that the average representation of one sample is equivalent to the average representation of another sample, as long as both samples originate from the same population. As such, researchers have often used one sample to generate the behavioral RDMs and a different sample to obtain the neuroimaging data. As generally valid (and in some cases necessary) as this method is, such an approach is not sensitive to individual-level information, the importance of which has been frequently addressed (17, 39–42), especially with respect to translational neuroscience (21, 43, 44).

On the other hand, recent studies have demonstrated that individualized RDMs can reveal meaningful individual-level information in cortical activity patterns (29, 45–47). Levine et al. explicitly compared the results between individualized emotion RDMs and a sample-averaged emotion RDM (29). This analysis yielded an interaction: effects based on the individualized RDMs were present in the insula but not in ventral temporal cortex, while the effects based on the averaged RDM were present in ventral temporal cortex but not in the insula. Such results suggest that the insula may be a common location for individuals' distinct emotion information: a finding that one cannot reveal when using a single RDM based on averaged behavioral data. This approach provides researchers the ability to interpret between-subject differences of neural RDMs [i.e., in the absence of second-order isomorphisms (38)] as meaningful individual differences in the underlying mental representations, which nevertheless share a common neural substrate (in this case, the insula).

Moreover, recent studies have shown that individuals' behavioral representational spaces correlate with other individualized factors [e.g., facial categorization with distractibility (46), semantics with fluid and crystallized intelligence (48), and affect with personality factors (49)]. Given these findings, it is likely that critical information is removed when averaging individuals' RDMs. Instead, by retaining such individualized information, these methods will benefit translational neuroscientists and clinicians in developing imaging-based biomarkers that address the cognitive level of mental disorders.

## Task-Congruency When Acquiring Behavioral and Neural Data

Behavioral similarity of concepts is task dependent. Recent work has seen participants perform the multi-arrangement task following different organizing principles; consequently, the underlying representational spaces differed as a function of the organizing principle that participants followed (29, 31, 50, 51). Such findings are in line with prior neuroimaging studies that have shown prominent changes in the neural representational space when participants engaged differently with the stimuli. For example, compared to a distractor task, a color-naming task increased the categorical nature of the representational space of colors underlying visual areas (52). Relatedly, having participants attend to either the behavior or the taxonomy of animals depicted in video clips shifted the representational space to emphasize the attended feature (8). Given these findings, having participants *not* attend to the features-of-interest (with which the behavioral RDMs were generated), for example *via* passive-viewing or distractor tasks, likely results in decreased sensitivity, as the neural RDMs contain a different underlying structure from the behavioral RDMs. Thus, such approaches uncover some form of automatic processing of the stimuli that is the least common denominator between the neural data and the behavioral data.

Additionally, one recent and intriguing finding came to light when different groups of participants were tasked with arranging a set of images according to their background, color, function, or shape (51), while a fifth group was instructed to freely arrange the images on the computer screen. The resulting averaged behavioral RDM from the “free-arrangement” sample resembled the behavioral RDM from the sample that arranged the images according to the similarity of their functions. This finding raises the question of what “default” information underlies representations in the absence of specific task demands, and how not controlling for this default processing may lead to neural representations that contain (possibly fluctuating) subjective biases toward the stimuli (53), thereby reducing the statistical power of the RSA. This issue is especially pronounced given that individuals have different attentional biases (54–58) and fluctuate between different mental states in an individualized manner (59–62). As such, during passive-viewing or unrelated tasks, the computational goal of the neural transfer function may not be sufficiently specified, resulting in the underlying structure of information present in the behavioral representational spaces differing from that in the neural representational spaces.

This approach still yields interesting group-level information regarding automatic processing or common attentional biases (and maintains a level of methodological simplicity by only needing to carry out one MR scan to which multiple distinct behavioral RDMs can be applied). However, since the nature of these automatic processes is undetermined, future studies that seek to investigate differential aspects of cognitive processes should consider controlling for mental operations during the scanning session. Addressing this issue will be especially pertinent to researchers and clinicians interested in applying such methods to patient populations, given the attentional and state-dependent biases of psychopathology (63–68).

## Possible Adaptations

### Consider Multiple Sessions Per Participant

One of the most straightforward ways to avoid needing to use averaged behavioral RDMs involves planning an experiment that includes RSA and the multi-arrangement method to consist of (at least) two sessions (i.e., one behavioral and one neuroimaging) per subject (29, 45). This way, every subject would produce at least one individualized behavioral RDM, allowing for an individuals' own behavioral data to be applied to their own neural data, thereby taking advantage of the individual-level information.

An additional consideration is that it is certainly possible that some representational spaces are more homogeneous across participants than others (e.g., perceived shape of a stimulus vs. emotion evoked by a stimulus). Relatedly, Hebart et al. recently revealed that similarity judgments between objects can be described by 49 dimensions (69). As such, depending on the dimension under investigation, using individualized RDMs may provide only minor benefits, possibly rendering the increased complexity of the experimental design unwarranted. Nevertheless, even slight differences in seemingly homogeneous representational spaces may be meaningful in particular research domains (e.g., color perception in synesthesia).

### Engage Participants With a Feature-Related Task

For the purposes of individual differences, addressing task-incongruency can involve having participants performing a task in the scanner that relates to the stimulus feature that also drove the organization of the stimuli during the behavioral session (or the theoretically motivated RDM). A step in this direction is seen in two recent studies that combined behavioral RDMs from the multi-arrangement method with fMRI: Bracci et al. asked participants to indicate whether a stimulus looked like (i.e., appearance condition) or depicted (i.e., animacy condition) an animal (70), and Tucciarelli et al. asked participants to indicate when they observed the same action on two consecutive trials (31).

Along these lines, the goal is to at least turn the participants' attention toward the features that underlie their related behavioral representational spaces. For example, if during the behavioral task participants organized pictures based on how the images made them feel, a simple task in the scanner could involve having participants press a button with their index finger if they like the picture presented to them and press a button with their middle finger if they dislike it (of course counterbalancing the stimulus-response mapping across experimental runs). A color-based task could ask participants to indicate if the colors in the stimulus tend to be brighter or darker; likewise a function-based task could ask participants to indicate if they use the object depicted in the stimulus on a regular basis. Although these tasks yield a binary outcome (while the behavioral data are continuous), participants are nevertheless turning their attention toward the same aspects of the stimuli as during the behavioral task, thereby ensuring an increased similarity between the computational goal of the neural transfer function of different participants, such that observed differences between participants' neural and behavioral RDMs should reflect related differences between their mental representations.

The downside to this approach is that scanning sessions would necessarily require more time, as one would need multiple in-scanner tasks to account for each behavioral model generated by the participants. However, this drawback may be warranted, if it results in higher sensitivity and accuracy of the analysis, which could improve the detection of individual-level information or permit subtyping of participants.

## Concluding Remarks

As representational similarity analysis is increasingly being used to investigate questions in cognitive and clinical neuroscience, it is necessary to address a few ways in which this method can be optimized for investigating both individual differences and commonalities in the context of individual differences. While the current approaches have revealed many interesting findings from a general or group-level perspective, we hold that taking advantage of individualized behavioral RDMs and controlling for task-related idiosyncrasies will ultimately allow for more informative neuroimaging investigations of differences between individuals' mental representations. These two methods work together toward this goal in that individualized behavioral RDMs permit the discovery of between-subject differences in mental representations, while task-congruency ensures that said differences can be interpreted within the context of the computational goal of individuals' neural transfer functions. Such an approach will allow researchers to test a variety of hypotheses that pertain to mental health disorders. For example, one could investigate whether individuals with emotion dysregulation have altered emotion spaces that are specific to particular brain regions, and, moreover, whether idiosyncrasies in these neural emotion spaces can assist in differential diagnosis of psychopathology, especially given the transdiagnostic nature of emotion dysregulation (71). Additionally, one could examine whether individual differences in behavioral and neural similarity of trigger stimuli are prognostic of treatment success in patients with anxiety disorders. As such, we find these methodological changes to be essential for optimal translation of task-based functional neuroimaging into fields where individual-level information is crucial, such as precision psychiatry.

## Data Availability Statement

The original contributions presented in the study are included in the article/supplementary material; further inquiries can be directed to the corresponding authors.

## Author Contributions

SL drafted the manuscript. Both authors devised the argumentation, revised, and approved the final version of the manuscript.

## Conflict of Interest

The authors declare that the research was conducted in the absence of any commercial or financial relationships that could be construed as a potential conflict of interest.

## Publisher's Note

All claims expressed in this article are solely those of the authors and do not necessarily represent those of their affiliated organizations, or those of the publisher, the editors and the reviewers. Any product that may be evaluated in this article, or claim that may be made by its manufacturer, is not guaranteed or endorsed by the publisher.
